# Parents’ expectations of staff in the early bonding process with their premature babies in the intensive care setting: a qualitative multicenter study with 60 parents

**DOI:** 10.1186/1471-2431-13-18

**Published:** 2013-02-01

**Authors:** Sonia Guillaume, Natacha Michelin, Elodie Amrani, Brigitte Benier, Xavier Durrmeyer, Sandra Lescure, Charlotte Bony, Claude Danan, Olivier Baud, Pierre-Henri Jarreau, Elodie Zana-Taïeb, Laurence Caeymaex

**Affiliations:** 1 Réanimation et Pédiatrie Néonatales, Hôpital Robert Debré, APHP, Paris, France; 2 PremUp, Paris, France; 3 Service de Médecine et Reanimation néonatales, Hôpital Port-Royal Cochin, APHP, Paris, France; 4 Service de Médecine Néonatale, Centre Hospitalier Intercommunal de Creteil, 40 avenue de Verdun, Créteil, France; 5 CRC CHI, Créteil, France; 6 Université Paris Descartes, Paris, France; 7 Université Paris-Diderot, Sorbonne Paris Cité. Département Hospitalo-Universitaire "PROTECT", Paris, France; 8 INSERM U676, Paris, France; 9 INSERM U669, Université Paris Descartes UMR-S0669, Paris, France

**Keywords:** Prematurity, Bond, Child development, Newborn, NICU, Nurses, Family centered care, Parenting

## Abstract

**Background:**

During the first weeks of hospitalization, premature babies and their parents encounter difficulties in establishing early bonds and interactions. Only a few studies have explored what caregivers can do to meet parents' needs in relation to these interactions and help optimize them. This study sought to explore parents' perception of these first interactions and to identify the actions of caregivers that help or hinder its development.

**Methods:**

Prospective study, qualitative discourse analysis of 60 face-to-face interviews conducted with 30 mothers and 30 fathers of infants born before 32 weeks of gestation (mean ± SD: 27 ± 2 weeks of gestational age), during their child's stay in one out of three NICUs in France. Interviews explored parental experience, from before birth up to the first month of life.

**Results:**

Data analysis uncovered two main themes, which were independent of parents' geographical or cultural origin but differed between mothers and fathers. First, fathers described the bond with their child as composed more of words and looks and involving distance, while mothers experienced the bond more physically. Secondly, two aspects of the caregivers' influence were decisive: nurses' caring attitude towards baby and parents, and their communication with parents, which reduced stress and made interactions with the baby possible. This communication appeared to be the locus of a supportive and fulfilling encounter between parents and caregivers that reinforced parents' perception of a developing bond.

**Conclusions:**

At birth and during the first weeks in the NICU, the creation of a bond between mothers and fathers and their premature baby is rooted in their relationship with the caregivers. Nurses' caring attitude and regular communication adapted to specific needs are perceived by parents as necessary preconditions for parents' interaction and development of a bond with their baby. These results might allow NICU staff to provide better support to parents and facilitate the emergence of a feeling of parenthood.

## Background

In premature babies, the quality of the early mother-child relationship influences subsequent development [1-5]. Prematurity *per se* does not necessarily affect the quality of mother-child interactions in the long term [6-9]. Maternal depression, on the other hand, has been identified as a risk factor for poor mother-infant interactions [6].

Mothers and fathers faced with their newborn's admission to the neonatal intensive care unit (NICU) endure a particularly difficult experience that may include parental anxiety, depression, and posttraumatic stress [10,11]. Factors contributing to the parents’ psychological state are their dependence on caregivers, the NICU's rules and organization, and uncertainty about the child's prognosis [12-14]. In addition, very preterm children and their parents have few opportunities to start developing a reciprocal relationship during the first weeks of life [15]. Finally, the father’s support to the mother appears essential to the latter [15].

These data indicate that NICU interventions aimed at restoring maternal self-esteem, reducing maternal stress and depression, and encouraging interactions with the child are an integral part of care necessary for very preterm newborns [3,16,17]. Programs to limit maternal stress [18], to create opportunities for parental empowerment [19], and to foster skin-to-skin contact [20], and changes in the physical layout of units [20,21] have all shown positive effects on the well-being of parents of infants in the NICU. Moreover, nurses in these neonatology units seem to play a key role in facilitating the start of positive mother-baby interactions [22,23].

Although reports have described the global experience and needs of mothers and fathers in NICUs, few studies have examined parental opinions about how caregivers can help them to interact positively with their babies and to perceive and present themselves as parents, and those have mainly used scales or questionnaires [16,24-26].

To better understand what caregivers can do to help mothers and fathers in their early interactions with their newborn in the NICU, we conducted an in-depth qualitative prospective multicenter study among parents of very preterm babies during their child's NICU stay. The objective of this study was to explore, through parents’ accounts, how an early bond with their very premature child is established and to identify their expectations of caregivers, and the concrete things that helped and hindered them.

## Methods

Face to face interviews with mothers and fathers of preterm newborns while the baby was in the NICU.

### Participants and procedure

The study took place at three tertiary care centers in the Paris region, France. All three were open to parents 24 hours a day and provided telephone contact. Visits to the baby were generally limited to the two parents. Meetings to provide news were organized regularly by the physicians. Parents were eligible for this study if they spoke French and if their child was born before 32 weeks of gestation, was 15 to 30 days old at inclusion, and had no recent severe clinical aggravation, according to the attending physician. The sample, defined in advance, comprised 30 fathers and 30 mothers, that is, 10 of each at each site. The nurses participating in the research (SG, NM, EA, CB, and BB) gave eligible parents an informational letter that described the study's objective, organization, and the use of the data for research and teaching purposes. The parents participated on a voluntary basis, making an appointment themselves with the interviewer.

### Data collection and analysis

#### Data collection

Semi-directive interviews lasting 60 to 90 minutes were conducted by a social psychologist trained inresearch and not involved in a NICU. Audio recordings of the interviews were made, with the parents' oral consent. Fathers and mothers were interviewed separately. The interview guide was developed from a review of the literature and from 10 preliminary interviews discussed within focus groups of caregivers, conducted by the researchers. During the interview, parents were encouraged to describe freely how they had experienced the news that the birth would be preterm, the actual birth, and the child's admission to and stay in the NICU. They were encouraged todevelop in detail everything that had helped or hindered them in developing a bond with their baby, as well as the activities set up by the caregivers (see the more detailed description of the interview outline in Additional file 1).

#### Data analysis

Discourse analysis was used to study the interviews [27]. The analysis was performed separately by a research psychologist and a research assistant. They conducted a horizontal analysis, with immersion and manual coding of the themes in each interview, and a vertical analysis that compared the themes throughout the corpus, identified the convergences and divergences of the same theme across different interviews and developed interpretative hypotheses. Particular attention was paid to the emergence of new themes and contradictory results as the interviews and analysis progressed and data saturation occurred.

The child's medical data and the parents' medical and sociodemographic data were collected from the medical files before and at the time of the interview.

### Ethics

This study was conducted as part of a Quality Improvement Process. The relevant Ethics Committee, the Ile de France 3 Committee for the Protection of Persons, approved the information provided, recruitment mechanism and type of consent, and their compliance with French laws and regulations. Processual consent was obtained throughout the interview [28].

## Results

Between November 2009 and March 2010, 68 parents were contacted, and 60 agreed to participate in the study — 30 fathers and 30 mothers, including 16 couples and 14 parents of each sex participating without their partner. The study included the parents of 49 children, born at a mean term of 27± 2 weeks of gestation, with a mean (±SD) birth weight of 956 (±206) grams. The children's mean (±SD) age at the interview was 24 (±10) days. The social and demographic characteristics of the participating parents and the clinical data about their children are presented in Table 1.

**Table 1:** **Characteristics of the study population** (**60 parents and their 49 children**)*

**Parents**' **characteristics**	**Mothers N**=**30**	**Fathers N**=**30**	**Total N**=**60**
Age (years), M±SD	30.7 +/− 6.6	33.5+/− 6.8	
Current professional activity			
Managerial and professional occupations	11 (37)	11 (37)	22 (37)
Skilled manual and non-manual occupations	18 (60)	15 (50)	33 (55)
Unemployed	1 ( 3)	4 (13)	5 (8)
Single parent	1 ( 3)	0 (0)	1 (2)
Parent’s place of birth			
Europe	12 (40)	16 (53)	28 (47)
Sub-Saharan Africa	10 (33)	6 (20)	16 (27)
North Africa	3 (10)	6 (20)	9 (15)
Asia or Mideast	2 (7)	0 (0)	2 (3)
Unknown)	3 (10)	2 (7)	5 (8)
History of preterm delivery	3 (10)	3 (10)	6 (10)
Assisted reproduction	6 (20)	5 (17)	11 (18)
Caesarean section	22 (73)	18 (60)	40 (68)
**Children**'**s characteristics at the time of the interview**			**Total N**=**49**
Female			29 (59)
Inborn			36 (74)
Multiple			12 (24)
Cranial ultrasound			
Normal			38 (77)
PVH-IVH grade 1 or 2			10 (20)
PVH-IVH grade 3			1 (2)
PVH-IVH grade 4, PVL			0 (0)
Ventilation at time of interview			
Spontaneous Ventilation			8 (16)
Nasal Ventilation			30 (61)
Endotracheal ventilation			11 (22)
History of Sepsis			23 (47)
History of NEC or GIP			3 ( 6)
Persistent Ductus Arteriosus, Surgery			11 (22)
Transfusion			30 (61)

PVH-IVH: periventricular-intraventricular hemorrhage (Papile’s classification), NEC: necrotizing enterocolitis, GIP: gastrointestinal perforation.

*all values are numbers with percentages in parentheses, except as otherwise stated.

The results are illustrated by direct quotations, followed by the designation m (for mother) or f (for father) and the interview number.

### Description of the feeling of an early bond by mothers and fathers

Parents reported that interactions with their child played a role in their feeling of being the child's parent and of having a specific bond with him/her.

The mothers consistently described three items that helped them to feel a bond with the child: physical closeness to the baby, increased knowledge with contact, and being able to leave the baby a toy or a fabric permeated with her odor. Most women insisted on their need for maternal gestures such as skin-to-skin contact or kisses: "*Me*, *the skin*-*to*-*skin*, *I loved it* … *feeling her*, *having her pressed against me*. *I said to myself*, *she*'*s really my baby*, *mine*, *today*. *I felt that I was her mother before the skin*-*to*-*skin*, *but there* … *that*'*s it*, *she was lying there*, *peaceful*" (m11). "*The hardest thing for me today*, *it*'*s the kisses*. *The fact that I cannot kiss her*, *because the bond with her*, *it*'*s all that*: *it*'*s playing with all her senses*, *as much as possible*, *it*'*s talking to her*, *touching her*, *being there*, *that she feels the love in our gestures*" (m15). Being close also implied knowing how to touch one's baby without hurting him/her.

A majority of fathers spontaneously reported that they agreed to carry their baby but preferred interaction by words and looks; few mentioned a physical need to carry her/him. A minority said they wanted to keep some physical distance from the baby, to keep from hurting him/her: "*Me*, *for the moment I film him*, *that*'*s enough now* (…). *I have stiff hands*, *I*'*m afraid of hurting him*, *he*'*s too little*. *It makes me happy to see my wife take care of him*, *for now*, *not me*!" (f9). Most of the fathers also reported that they interacted with the baby to promote their wife's psychological well-being: "*My wife is very happy that I do skin*-*to*-*skin because my father never took care of me*, *and she is really worried that I will be the same*" (f5). Finally, breastfeeding was considered very important by fathers and breastfeeding-mothers in feeling a bond with their child, but the mothers described the numerous problems in pumping their milk and their frustration about the delay between its collection and its administration to the baby.

### The nurses’ influence on early interactions and on parents' personal perception and presentation as the father/mother

#### The nurses' caring qualities toward the child and the parents

Most parents described themselves as dependent on the staff to care for their baby and therefore necessarily subject to its authority: "*As we are in a place where everything is managed by others and we don*'*t know*, *we have the impression that we have to ask for permission to touch him*" (m17). Nearly all the parents reported that the caring and considerate conduct of the staff was primordial for the interactions they developed with their child. Both mothers and fathers reported that a gentle attitude towards their child attenuated the strangeness of the intensive care unit: "*There are extraordinary nurses*, *they talk all the time to the baby*, '*my sweetheart*, *I*'*m going to do this for you*.' *I love it that she is in such good hands*" (m13). Parents described their ability to have contact with the baby linked to the nurses' conduct, because it made the contact possible (or not) and pleasant (or not). Most parents reported a good relationship with the nurses, who anticipated their expectations and had a gentle attitude toward both parents and baby: "*There are disparities between the nurses in terms of their characters and their ways of seeing things*. *Some are very affectionate with the babies*, *they give parents lots of information*. *It*'*s great because it creates a human bond*" (m5). A gentle attitude towards the child when providing care encouraged the parents to stay close. "*The nurse who talks to my daughter and doesn*'*t make brusque gestures* … *that makes us want to stay*" (m5). A minority stressed more distant and colder nurses: "*There are others*, *there*'*s not much of a relationship*. *They wait for us to ask questions*, *and their answers are very terse*" (m5). Some added that it felt appropriate to take moments of closeness to their child only when the nurse was kind enough to offer them. Some mothers reported feeling judged by the nurses: "*I have the impression of being judged if I call too often* … *and if we don*'*t come*, *I*'*m also afraid of being judged*" (m8). They therefore behave differently than they would spontaneously. Globally, mothers described themselves as simultaneously dependent on and close to the nurses, while fathers commented more on the nurses’ behavior towards their children and wives than towards themselves.

#### Communication

Communication with the caregivers appeared decisive for fathers and mothers in feeling a bond with their child. Communication included the content of information exchanged, but also the relationship that existed in those moments. Globally, the consistency of communication was always reported as essential. Throughout the stages from the prenatal period to the first weeks in the NICU, mothers and fathers described expectations that were often similar, and sometimes different. Some fathers reported that the staff spoke to them less than to the mother, which seemed normal or more rarely, frustrating in their role of father.

- In the prenatal period, an exchange with a neonatologist, mentioning the baby's approximate weight and height and a possible relationship with him, triggered the first perception of themselves as parents — father or mother: "*The pediatrician told me that he knew how to take care of this baby and that opened a door to the future because two weeks before*, *the ultrasonographer had told me to separate myself from him*" (m3). On the other hand, the existence of discordant information about the thresholds for resuscitating preterm babies was described as very upsetting: not knowing if the child would live or not prevented parents from perceiving themselves as such.

- In the delivery room, mothers reported that they had needed explicit communication — words — about the baby's health, to be reassured that he was really alive: "*As soon as I woke up*, *I asked*: *He*'*s not dead*? *He*'*s not dead*?’ *They told me right away that my son was alive*" (m19). Seeing the child, even briefly, was also described as an occasion of feeling like a mother. The fathers talked about the delivery as a moment of intense stress with simultaneous worry for their child and their wife: "*Paradoxically*, *I was more worried for my wife than for my daughter*" (f14). Several reported not having been able to feel close to their baby until they had been reassured about their wife's health: "*Just after the birth*, *I went to see my baby with the pediatrician* (.) *My wife*, *I was thinking of my wife*. *It was hard for the baby but I saw him without really appreciating it*" (f18).

- After the delivery, many mothers reported having had to wait a day or two before being authorized to see their baby, for health reasons. The photograph of the baby and the NICU caregivers' visit to the mother's room were the two factors described as very useful for feeling closer to the child in these cases. Almost all the parents reported having received a photograph in the first hours after birth and they appreciated it: "*It was good to have this picture*. *I had two feelings*…*I was glad and sad at the same time*…*sad because she was premature*" (m9). The fathers saw it especially as helpful for their wife, and indirectly for themselves: "I *would have liked my wife to have had a picture from the beginning because for the mothers who cannot see their babies*, *it*'*s the best solution*. *She*'*s the mother*, *she needs to see her baby*. *I reassured her as much as I could*" (f11). They also mentioned the photograph as the evidence of a humane attitude by the caregivers: "*I thought that was nice*, *it showed the mind*-*set of the nurses*; *I understood then that they didn*'*t care only about the medical care but also about the attachment between parents and child*" (f2). The quality of the photograph had to be good, otherwise it would reinforce the harshness of the preterm birth. Several parents described their poor experience with caregivers who promised a photograph that they finally never gave: this unkept pledge reinforced the absence for mothers and created a feeling of impotence in fathers: "*I was a little disappointed because a midwife had told me a photo would be taken*, *and that never happened*. *I think that would have helped me*, *if it had been printed right away*" (m4). Both parents also reported the supportive value of a visit by the pediatrician or the nurse to the mother's room, telling them about the baby's health. Some mothers saw it as the occasion of their first meeting with the NICU team: "*For 3 days I wasn*'*t able to see my daughter*. *The doctors came to see me and the nurse also*. *I found that encouraging*: *I was very glad to get news about her*. *The information was clear*; *they told me that she is small but doing well*. *She has a catheter*, *and a feeding tube for eating*. *Otherwise*, *we know she*'*s alive*, *but we don*'*t know how she*'*s doing*, *it*'*s just total darkness*. *Their coming cheers me up*, *and that night I slept well*. *Saying to myself that she*'*s doing well*" (m11). On the other hand, the absence of such visits was described by many mothers as a source of isolation and stress and an obstacle to their self-representation as mothers. Many mothers said that they were frustrated to have to rely on the child's father for new information: "*It would have been good if someone from the team had come down to see me*, *because my husband is not a physician*. *If the NICU nurse had come and said to me*, "*I*'*m the one who saw her first*, *I did this to her*. *Had she come for two minutes*, *that would have reassured me still more because they are the ones caring for my baby*" (m5).

- Most mothers and fathers needed a caregiver to receive them for the first visit to the NICU, perceived as very stressful: "*You come into this grim place*; *you need someone to say to you*, '*you*'*ll see*, *it*'*s a little strange*, *a little dark*, *but that*'*s normal*,' *because at the beginning*, *what*'*s dark*, *is death*" (m28). The fathers accompanied their child from the delivery room but frequently described an anxious wait at the ward entrance: "*I would have liked it*, *when I arrived in the unit*, *for someone to come out and say to me*, '*Your daughter is in good hands*, *we are going to take care of her*‘, *just to reassure me that everything was all right*. *And then I would have had some news for my wife*; *I didn*'*t have that*" (f23). Many mothers mentioned their difficulties in reporting what had been said at their first visit, but the attention of the caregiver, perceived by her presence and words, had reassured them: "*I don*'*t remember very well what the nurse said to me*, *but I know she said*, *don*'*t worry if that goes off*. *I admit*, *I*'*ve forgotten a lot*, *it was a very particular moment*" (m22). They mentioned that it had been important to them that the nurse introduced herself by her first name — "*I*'*m X*, *and I*'*m taking care of your baby*" — with a pleasant attitude. Fathers, on the other hand, remembered the information quite clearly.

- In the first weeks in the NICU, access to regular explanations helped most of the parents to limit their feelings of helplessness and to be able to come see the baby day after day. To be at ease with their child, the parents reported that they needed to understand the environment: "*The more I know*, *the more I am reassured*. *What I want to know are the upper and lower limits*, *because I watch the monitor and I have the impression I understand*" (f23). The mothers saw a need to be reassured when they were holding their child: "*At the beginning of skin*-*to*-*skin*, *I didn*'*t really know what was going on*: *when it rings*, *for us*, *that*'*s disastrous*, *so if you don*'*t see someone come*, *you say to yourself*, *but what*'*s going on*? *In fact*, *there are alarms that are not so serious*, *but they hadn*'*t told me*, *so it would be good if they said at the beginning what it means*" (m11).

The mothers said more frequently than the fathers that they needed explanations of the baby's relational capacities and on the meaning of their reactions, to help them: "*It*'*s important to understand her reactions*, *when she cries or seems nervous*. *If I don*'*t manage to calm her*, *I feel like a bad mother who does not understand her child*. *It*'*s important to understand and also to know what to do next*" (m15). Fathers and mothers both insisted on the need to warn them of changes such as intubation, changing the room, or placing a catheter: "*I arrive*, *there are 3 physicians in the room*, *a blue sheet over my baby*. *And there*, *I panic*! *The doctors say to me*, "*You can*'*t come in*." *I say to myself*, *they*'*ve put this sheet so that I can*'*t see*… *They*'*re taking her to the morgue*. *Fortunately*, *there was someone who saw that I was stunned and explained to me*: *They*'*re changing the catheter*‘" (m30). Any situation that was unexpected or not understood created a feeling of panic and kept them from spending peaceful time with the baby. They also described their need to not be kept waiting about exam results, such as ultrasound: "*If there is no problem with the examinations*, *the doctors don*'*t come to tell you the results*. (.) *If they tell us the results right away*, *whether they are good or bad*, *we know them and we can start to enjoy the child*" (m18).

Some mothers described a feeling of moving backwards in their bond with the baby when his condition grew worse: "*What is a little frustrating in the relationship*, *it*'*s that I had the impression that there was more interaction at the beginning than now*, *because she has an infection so she isn*'*t very responsive*, *while before*, *she took my hand*. *I arrive*, *she is grumpy*, *she cries*, *so I don*'*t know if it*'*s something to do with me* … *so I say to myself*, ″*Oh*! *I*'*m not going to come anymore*" (m13). The telephone was described as a way of staying linked to the baby from home. Most parents reported feeling reassured by ritualized calls morning and evening: "*It*'*s very good to have news by telephone*… *it takes 15 seconds but afterwards*, *you feel so much better*… *then pffff*! *I pump my milk and I fill the bottle*" (m7). Some described calls more worrisome than reassuring, in cases where the phone rang repeatedly with no answer, and stressed the importance of always giving news, even succinctly. Most parents preferred to go home at night rather than stay in a room at the hospital, because it helped them to regain their bearings.

The caregivers’ actions reported by parents as useful for early bonding with their preterm newborn are listed in Table 2.

**Table 2:** Caregivers’ actions reported by parents as useful for early bonding with their preterm newborn


Before birth	Prenatal visit by neonatologist
	Explanations: baby’s weight and capacities, breastfeeding, child's course
	Introduction of staff and department operation
	Consistency of descriptions of management
At birth	Briefly reassure parents about baby's condition (alive)
	Reassure father about the mother's condition
Between delivery and first visit	Visit mother in her room
	Provide photograph of the baby
	Interact directly with both parents, especially if clinical status of the baby is worsening
In the NICU	Accompany each parent on first visit
	Gentle and attentive attitude toward the baby
	Explanations: machines, alarms, child's capacities and ways of helping him
	Help parents become more autonomous progressively, gently and kindly, avoid judgments
	Suggest closeness: carrying the baby, skin-to-skin (anticipate and support these moments)

## Discussion

The objective of this study was to explore in detail how preterm neonates’ fathers and mothers describe the early bond with their child in the first weeks of life and to identify the steps caregivers can take to meet parents’ expectations. Globally, fathers and mothers described interactions with their child as important steps in starting to feel a bond with him/her. Fathers did not interact with their baby in the same way as mothers, and they described different expectations towards the staff. Both parents however expressed the need for communication with caregivers before birth, at delivery, and in the NICU.

Our study found that receiving explanations about the baby’s health status, how the NICU and its equipment operate, and warning about changes were very important for parents to be able to spend feel at ease and thus able to bond with their newborn. Parents made it quite clear that fear of death and anxiety impeded their ability to interact with the baby. Other studies with parents have described periods of panic, depression or joy according to the baby's health status [13,29]. Parents need reassurance to concentrate on their baby and professional assistance to interact with him/her with a sense of security.

Parents reported that explanations of the baby's sensory and relational capacities and of possible helpful activities were essential for meeting their child and for feeling that they were indeed the baby's parents. This is in agreement with other studies, which highlighted the difficulty in interpreting premature newborns’ reactions [2], described parents' subsequent feelings of incompetence in caring for them [30], and the importance of information about the infants' reactions, to combat this negative parental feeling [31].

An important finding of our study is that parents expect these explanations to be transmitted through a caring, humane attitude of exchanges with them, rather than as technical knowledge. This was suggested by Lupton and Fenwick’s finding that "chatting" was important to establish family centered care in the NICU [23,32]. Watching caregivers who address the child with gentleness also helps parents move closer toward the baby they perceive as a tiny and fragile being. Our study suggests that the caregivers' consideration reinforces the fathers and mothers in their role and promotes the emergence of a feeling of being useful, good and irreplaceable for the child. This expertise approaches the concept of family centered care [33]: taking good care of the child is taking good care of the mother, and vice versa.

Our study also observed that fathers interact with their baby differently than mothers do, sometimes staying off to the side: for fathers, the relationship with their baby appeared less based on physical contact. The relationship is also marked by the need to understand machines and treatments, perhaps, as previously reported for fathers [34], to feel some control over the situation. In the first weeks, the mothers’ self-esteem and capacity to interact with their baby were rooted in their relationship with the nurses; the nurses' conduct could compensate for, or on the contrary feed into, a feeling of poor self-esteem. Fathers did not feel as dependent on nurses and intervened very little between their wives and the nurses. Mothers conserved especially a memory of the emotions perceived at the time of the communication and found it more difficult than fathers to remember the content of information transmitted [15]. Finally, many fathers also interacted with their child in part as support and reassurance for the mother. The fathers in our study also found it stressful to be unable to be with their partner and their infant; this concern was not mentioned by men in another study [15,35].

Our study did not show differences in the parents' expectations according to either their social and demographic characteristics or the child's clinical data. It is possible that needs become more homogeneous in this extreme situation — parenthood in the NICU [13].

An important strength of our study is the equal proportion of fathers and mothers, quite rare in the literature [16], which makes it possible to compare the needs of each group. Another strength is the representation of parents of non-European origin, who account for approximately half the participants; even if their French language skills indicate a degree of integration into French society, their participation and viewpoints add cultural perspectives often missing from this type of qualitative study.

Our study also has limitations: First, the timing of the interviews, at between 14 and 35 days of hospitalization, made it possible to direct messages to caregivers more specifically but might also explain that parents describe few attitudes of active parenting, a capacity that develops over time [11,15,29]. Secondly, only a few negative aspects of the child's care were reported in our study, possibly because parents felt less free to criticize because their child was still in the institution [36], even though the interviewer was independent. Thirdly, clinical characteristics of the participants’ children limit the generalizability of our results to families with fairly normal development patterns in the first month; parents of children with life-threatening complications might have different specific needs. Finally, the study took place in a limited geographic zone, and its results might not be transposable to other settings.

The qualitative method allows us to accede to the parents' point of view, and may thus provide information that goes beyond caregivers' stereotypes of parents and increase the former's empathy for the latter. One objective of NICU caregivers is to allow parents to become progressively more autonomous. Help in creating early interactions with the child and attributing value to the parents' role are essential prerequisites to this increasing autonomy. All caregivers can improve their professionalism by learning the concrete actions that can help to repair the psychological injuries to parents due to the delivery of a preterm baby [37].

Future research might concern the longitudinal experience of a sample of parents, at different times as they parent their premature child.

## Conclusions

Nurses' caring attitudes and caregivers’ regular communications, adapted to needs at different times, help parents to interact and create a bond with their premature neonate. These results might enable NICU staff to provide better support to parents.

## Abbreviation

(NICU): Neonatal Intensive Care Unit.

## Competing interests

The authors (SG, NM, EA, BB, XD, SL, CB, CD, OB, PHJ, EZT, LC) declare they have no competing interests.

## Authors’ contributions

Conceived and designed the study: SL, EZT, NM, SG, EA, XD, CB, CD, LC. Performed the study: SG, NM, EA, BB, CB. Analyzed the data: SG, NM, EA, BB, XD, EZT, LC. Wrote the paper: SG, NM, EA, EZT, BB, XD, CB, CD, OB, PHJ, LC. Participated in data analysis and interpretation: SG, NM, EA, BB, XD, EZT, LC. All authors have read and approved the final manuscript.

## Pre-publication history

The pre-publication history for this paper can be accessed here:

http://www.biomedcentral.com/1471-2431/13/18/prepub

## Supplementary Material

Additional file 1 **Interview guide.** Bonding between parents and their preterm child. (PDF 65 kb) Click here for file
