# Supplementary material for: Parents’ expectations of staff in the early bonding process with their premature babies in the intensive care setting: a qualitative multicenter study with 60 parents
Source: BMC Pediatr. 2013 Feb 1;13:18. doi: 10.1186/1471-2431-13-18 (PMC3568058; doi:10.1186/1471-2431-13-18)
Supplement: Additional file 1 — Interview guide. Bonding between parents and their preterm child. (PDF 65 kb) [file 1471-2431-13-18-S1.pdf]

ADDITIONAL FILE

Interview guide: Bonding between parents and their preterm child

Study presentation to the participant

Objective: Improve the researchers' understanding of the construction of the bond between mother or father and their preterm child in the first weeks in the NICU and how caregivers can help in this construction. The objective is to improve the caregivers' awareness of these issues and improve practices.

Interview conditions**:** audio-recording, use for training and scientific research purposes only: absolutely no use for commercial purposes or any internet distribution; anonymization of data; freedom of expression.

Condition: Oral consent for the research interview and for the use of the data and processual consent obtained.

Interview

The interview takes place in 4 parts, identifying the positive and negative points of each stage and the parents' suggestions for the caregivers, to respond concretely to their expectations.

Part 1: Free narrative of the pregnancy, delivery and postnatal period

Part 2: Detailed exploration of aspects of the parent-child relationship, seeking in particular the items that resulted in dissatisfaction or regret. Free narrative, and then questions if needed to obtain more details about the participants' experience: *Before the birth, relationships with healthcare staff and department, organization and experience of visits*

Part 3: Exploration of the construction of a bond with the baby: *First contact with the baby and possible communication with him, course of this bond*

Part 4: Expectations of the participants and concrete means of facilitating the creation of a bond between the parents and the preterm child.

Acknowledgments
